# Intestinal necrosis cannot be neglected in a patient with hepatic portal vein gas combined with appendicitis: a rare case report and literature review

**DOI:** 10.1186/s12893-019-0478-8

**Published:** 2019-02-04

**Authors:** Haimin Chen, Qingsong Wu, Hongcai Fang, Bo Liang, Lu Fang

**Affiliations:** 1 Department of Hepatobiliary Surgery, the Second Affiliated Hospital, Nanchang University, No. 1, mingde road, Nanchang, Jiang xi China; 2 Department of Hepatobiliary Surgery, Yuebei people‘s hospital, Shaoguan, Guangdong China; 3 Department of Hepatobiliary Surgery, Affiliated Hospital of Jiujiang University, Jiujiang, Jiang xi China

**Keywords:** Venous, Appendicitis, Intestinal necrosis, Abdominal pain

## Abstract

**Background:**

Hepatic portal vein gas (HPVG) is a rare acute abdomen, which is not an independent disease. Meanwhile, HPVG combined with appendicitis has been rarely reported. We found only a similar report by looking for literature, but no intestinal necrosis occurred. We report a patient with HPVG, appendicitis and intestinal necrosis was reported in the current study. The patient was given frequent monitoring and had been conducted operation in time.

**Case presentation:**

An 86-year-old female with appendicitis complicated by HPVG was reported in the present study. Abdominal examination revealed rebound tenderness at the McBurney’s point. Moreover, abdominal computed tomography (CT) revealed gas in portal and mesenteric veins in addition to appendicitis. An emergency operation was planned on the appendix. However, the patient refused surgical treatment. Therefore, conservative treatment of antibiotics and frequent imaging observation was conducted for this patient. Although imaging results suggested disappeared gas in intra- and extra-hepatic portal veins, the small intestine was dilated, after conservative treatment of antibiotics. In addition, signs of diffused peritonitis could also be observed and an exploratory laparotomy was performed. Intra-operative findings had confirmed suppurated appendix, mesenteric ischemia and small intestinal necrosis.

**Conclusions:**

Frequent monitoring benefits us in observing the progress of intestinal diseases. When there exist other possible causes of HPVG such as infection, it is not easy for us to ignore the possibility of intestinal necrosis.

## Background

Hepatic portal vein embolization often occurs in thrombus caused by cirrhotic portal hypertension, abdominal infection and abnormal coagulation function. In addition, it can also be resulted from cancer embolus caused by liver and pancreas tumors. However, HPVG is extremely rare. HPVG is characterized by acute onset, rapid progression, poor prognosis and high fatality rate. Meanwhile, it frequently occurs in intestinal ischemia and necrosis. The necessity of surgery and operation timing are the most crucial for HPVG. A patient with HPVG, appendicitis and intestinal necrosis was reported in the current study. The patient was given frequent monitoring and had been conducted operation in time.

## Case presentation

**Fig. 1:**
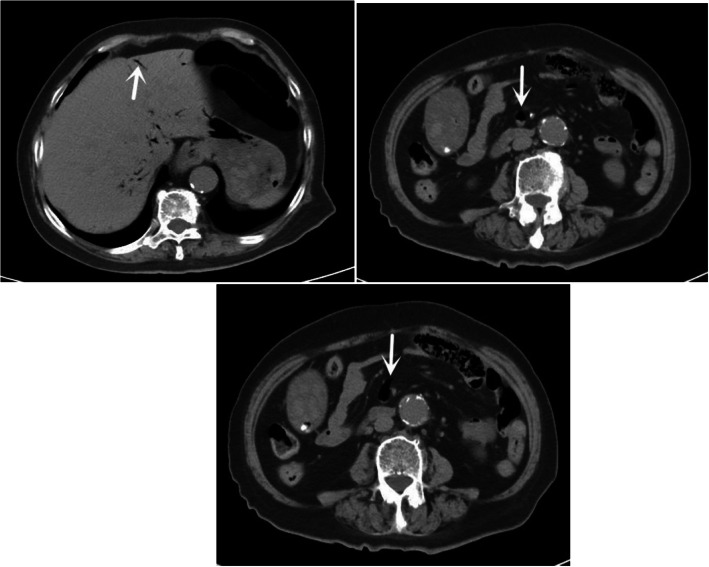
Axial section of abdominal computerized tomography on admission showed gas within the hepatic portal veins and the superior mesenteric vein (white arrows)

**Fig. 2:**
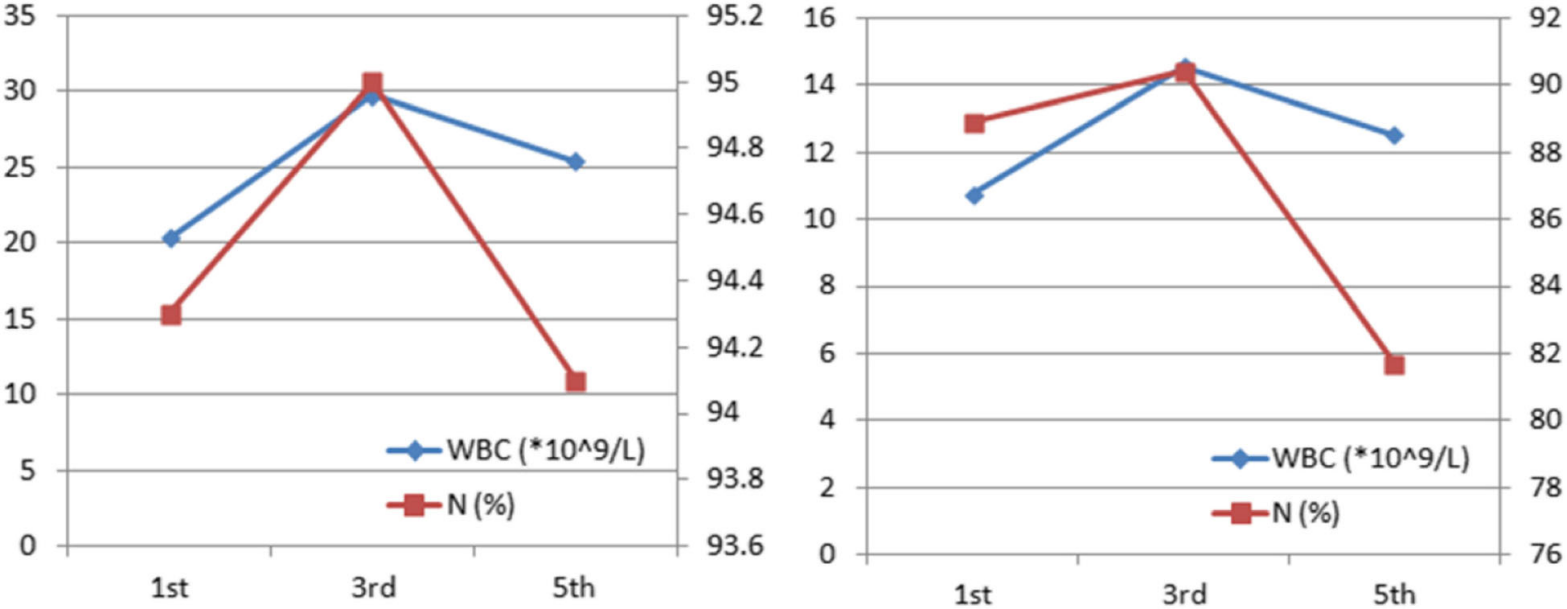
Preoperative (left) and postoperative (right) White blood cell (WBC) and neutrophilic granulocyte percentage (N%) changes in patients (the detection methods we used: electrical impedance for WBC and chemical staining for N%)

**Fig. 3:**
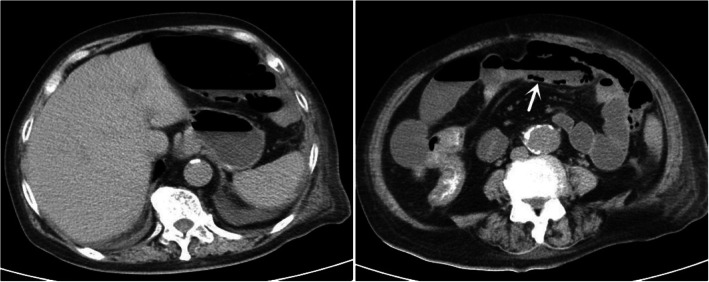
Axial section of abdominal computerized tomography on the third day after admission revealed remarkably absorbed gas in hepatic portal veins and superior mesenteric vein, pneumatosis intestinalis (white arrows), as well as small intestinal expansion

**Fig. 4:**
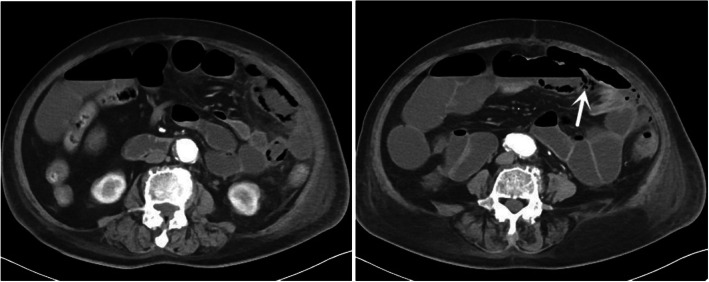
Axial section of abdominal computerized tomography on the fifth day after admission suggested exacerbated small intestinal dilation and pneumatosis intestinalis (white arrows)

An 86-year-old female consulted in our hospital as a result of aggravated lower abdominal pain for 6 days, accompanying with nausea, vomiting and diarrhea. The patient had a past history of hypertension, which was treated with amlodipine besylate tablets. Abdominal examination revealed rebound tenderness at the McBurney’s point, accompanying with normal bowel sounds. Laboratory examination of the patient on admission suggested white blood cell (WBC) count of 20.31*10^9/L and neutrophilic granulocyte percentage (N%) of 94.3%. Abdominal computed tomography (CT) revealed gas in intra- and extra-hepatic portal and mesenteric veins in addition to appendicitis with peripheral peritonitis (Fig. 1). Thus, an emergency laparoscopic appendectomy was planned, which was rejected by this patient. Therefore, conservative treatment with antibiotics (Piperacillin Sodium and Tazobactam Sodium for Injection) was applied. The abdominal pain was generalized for the following 4 days; moreover, the signs of diffused peritonitis and borborygmus had gradually disappeared. Nonetheless, no decreases could be seen in inflammatory markers after conservative treatment with antibiotics (Fig. 2). Subsequently, repeated abdominal CT scan was conducted, which revealed the absence of gas in intra- and extra-hepatic portal and superior mesenteric veins. However, abdominal CT scan revealed enhanced pneumatosis cystoides intestinalis and dilated small intestine, which had become more and more severe. In addition, the appendicitis with peripheral peritonitis had been always present (Figs. 3 and 4).

**Fig. 5:**
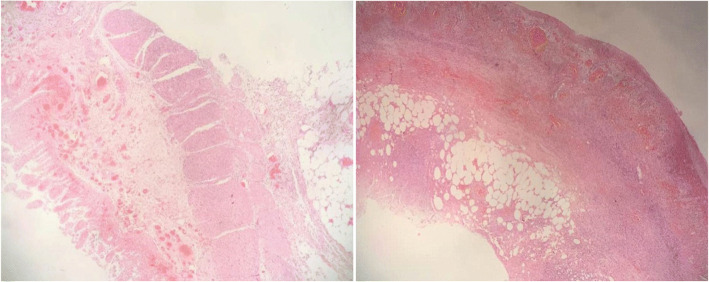
Pathological examination showed necrosis of ileum (left) and acute gangrenous appendicitis (right)

**Fig. 6:**
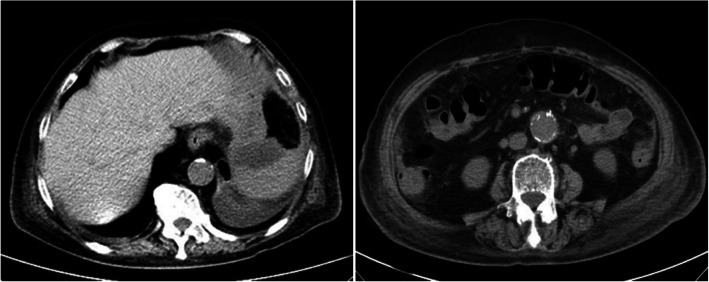
Axial section of abdominal computerized tomography on the fifth day after the operation and it indicated that the intra-abdominal condition was improved

Intestinal necrosis was suspected based on the abdominal signs, as well as changes of laboratory and frequent imaging examination results. An emergency laparotomy was performed on the fifth day of admission. Intra-operative findings had confirmed the diagnosis of suppurated appendix, with about 20 ml purulent secretion around it. Remarkable necrosis, which was about 100 cm, could be observed in the small intestinal wall and mesentery. Besides, an intestinal perforation could be seen in the necrotic bowel. The intestinal perforation was covered by the omentum majus. No obvious intestinal content overflow was seen. The patient had received appendectomy and part of the small intestine was removed. Afterwards, the patient’s postoperative pathology had confirmed the diagnosis of acute gangrenous appendicitis accompanying with periappendicitis and ileum necrosis **(**Fig. 5). Based on the use of antibiotics (Imipenem and Cilastatin Sodium for Injection), the inflammatory markers decreased significantly after surgery (Fig. 2). In addition, abdominal CT was performed on the fifth day after the operation, showing that the intra-abdominal condition was improved (Fig. 6). Her symptoms were resolved and she was discharged 11 days postoperatively.

## Discussion and conclusion

HPVG is a rare acute abdomen, which is often regarded as an ominous imaging finding. The most serious concomitant symptoms include intestinal ischemia and necrosis, which are the leading lethal etiological factors. HPVG was first described by Wolfe and Evans in the newborn subsequently dying of necrotizing enterocolitis in 1955 [1]. In 1960, Susman first reported the adult death case of HPVG [2]. Furthermore, the first survival case of HPVG was reported in 1965 [3]. HPVG is often reported as a sporadic case in literatures. It is associated with abundant etiological factors. Particularly, intestinal ischemia and necrosis, inflammatory bowel disease, gastric ulcer, digestive tract expansion, nephropyelitis, cholangitis, pancreatitis, intra-abdominal abscess, ovarian abscess, and iatrogenic causes (such as endoscopic surgery [4]), are all correlated with HPVG. Moreover, a post-operative patient with HPVG of no identifiable cause has also been reported [5]. However, HPVG complicated by appendicitis is seldom reported in relevant literatures.

Early diagnosis and treatment are important measures to effectively reduce the mortality of HPVG [4]. HPVG has been considered as a rare and dangerous label for signal transmission. It is associated with high risk and high mortality. Auxiliary diagnostic equipment has been constantly improved with the continuous development of medical technologies. Plain radiograph, ultrasound and CT have played certain roles in diagnosing HPVG currently [6, 7]. Plain radiograph: Portal vein belongs to centrifugal flow, which deforms from the first porta hepatis to the liver edge. It appears as linear and/or branching lucencies and displays the tiny branch of portal vein. Color Doppler ultrasound: Dotted strong echo can be observed in the portal vein lumen. HPVG can arrive in the liver edge or within the Glisson’s capsule along with the fast moving blood flow direction. CT: Gas in hepatic portal vein is transported to the liver edge along with the blood flow. It appears as linear or branching image in the tiny branch of portal vein [8]. It mainly presents in the liver parenchyma within 2 cm of the Glisson’s capsule, especially in the case of left lobe [4, 9]. Gas within the intrahepatic biliary channels often locates in the central liver parenchyma. Both of them can be distinguished on this basis [10]. Gas within mesenteric vein is tubular; alternatively, it locates in the edge of mesentery, which is branch-like. Early studies have shown that the mortality of HPVG is up to 75% [11]. Research on the high mortality is largely conducted based on the diagnosis of plain abdominal radiographs. The number of diagnosed cases is increasing, which can be attributed to the development and application of color Doppler ultrasound and CT with high resolution. Nelson research had demonstrated that the mortality of HPVG had reduced to 29–39% [12]. Thus, the mortality of HPVG patients has decreased due to early detection, early intervention and understanding of etiological factors. CT displays high sensitivity in diagnosing portal vein gas, which contributes to further understanding of the intestinal tract situations. Notably, reinforced CT can be employ to distinguish portal vein gas that can hardly be distinguished.

Three hypotheses are available regarding the cause of HPVG. To be specific, firstly, infection: gas-producing bacteria, such as *Clostridium perfringens*, *Escherichia coli* and *Klebsiella pneumoniae*, intrude into the intestinal wall or portal vein, which have bred, thus generating gas [6, 13]. Secondly, intestinal mucosal injury and increased enteric cavity pressure: intestinal gas can act under the action of increasing intestinal pressure in the presence of intestinal mucosal injury, which can thereby enter the portal vein system through the damaged mucosa [14]. Thirdly, mixed type: HPVG is the consequence of interaction of infection with mucosal injury [15]. In some cases, the intestinal mucosa is damaged, which fails to recover in a short time. Thus, the intestinal mucosa will develop secondary infection under the action of bacteria. Each etiological factor can act independently. Alternatively, they may also be mixed to produce the portal vein gas, which can lead to different consequences. Mitsuyoshi [16] studied cases with HPVG based on histology. He indicated that aerogenic bacteria would penetrate into the intestinal wall to damage the muscular layer in the presence of severe infection, which thus gave rise to pneumatosis cystoides intestinalis. Avascular necrosis is likely to take place in the intestinal wall; meanwhile, it is easy to form perforation. Thus, urgent operative treatment is often needed. Mechanical factors-induced mucosal injury is not accompanying with infection. Therefore, it is associated with low occurrence probability of intestinal perforation. In this case, intervention in surgery is not the preferred method. Koami [17] had proved that pneumatosis cystoides intestinalis was the independent risk factor of intestinal necrosis. Peritonitis severity is also considered as an available indication of urgent surgery. Tan [18] had reported patients with portal vein gas caused by enterocolitis after chemotherapy. The patients were conducted abdominal laparotomy in the absence of sign of peritonitis, but no intestinal ischemia or necrosis was observed. Fujikawa [19] had reported a patient with portal vein gas, who was given conservative antibiotics therapy in the absence of peritonitis. The portal vein gas disappeared subsequently, with no intestinal necrosis being seen. In addition, it is proved in some studies that, low systolic pressure, high-level lactic dehydrogenase [17] and increased lactate level with anion gap [12] are also risk factors of intestinal necrosis. In the current study, our patient may develop portal vein gas in the presence of serious infection. Conservative antibiotics treatment has been carried out, but the inflammatory indicators and abdominal signs can not be effectively controlled. What’s worse, the range of peritonitis range is increasing. Subsequent CT re-examination reveals pneumatosis cystoides intestinalis. The urgent exploratory laparotomy is thereby conducted on this basis. Intra-operative findings reveal partial necrosis in ileum, along with intestinal canal perforation.

The course of intestinal necrosis in HPVG patients with appendicitis has been analyzed in the current study. In our case, the initial symptom in the patient includes uncomfortable right lower abdominal pain, which is gradually aggravated. Thus, the patient consults in the hospital for treatment. Remarkable sign of peritonitis in right lower abdomen can be found on admission, along with normal bowel sounds. Laboratory inspection reveals high levels of WBC and neutrophil granulocyte percentage. Gas in the portal vein and mesentery has disappeared after conservative antibiotics therapy. However, the inflammatory indicators have not been notably improved, which maintain at high levels. Frequent imaging observation is performed, since the patient’s abdominal sign and laboratory inspection results have not been greatly improved. The results suggest that the patient has developed intestinal wall gas and intensified small intestine expansion, which is constantly intensified. Moreover, the range of abdominal peritonitis has also been expanded gradually. Under such circumstance, abdominal laparotomy is given in time. Erosion and suppuration of appendix can be observed in our patient during operation. The purulent secretion is about 20 ml around the appendix. In addition, obvious necrosis can be seen in the wall of ileum, along with a bowel perforation. Appendectomy and ablation of necrotic intestine are conducted in the patient, and antibiotics therapy is continued after operation. Afterwards, the patient is cured and discharged. The disease progression of the patient is analyzed, and she is confirmed to be attacked by acute appendicitis. HPVG and mesenteric vein gas is developed due to infection. Nonetheless, the causes of bowel necrosis can not be defined yet. In addition, HPVG and superior mesenteric vein gas may be caused by the interaction between appendicitis and bowel necrosis. Diana [20] had reported a patient with acute appendicitis complicated by HPVG, and the complications of retroperitoneal abscess and rectal perforation were also present. In this case, HPVG may be caused by the interaction between acute appendicitis and bowel perforation. As is reported in some literatures [21], no intestinal ischemia or intestinal necrosis can be seen intraoperatively in a patient with acute appendicitis combined with HPVG. It is noteworthy based on this case that, it is inadvisable to suspect appendicitis-induced HPVG only, while ignoring the other concurrent causes of HPVG such as intestinal ischemia and intestinal necrosis, since it will lead to severe consequence.

The high mortality of HPVG is considered as a life-threatening sign in the past, requiring an urgent abdominal laparotomy. Increasing cases treated with conservative treatment for HPVG have been reported as growing cases have been discovered. However, we still can not easily ignore the possibility of intestinal necrosis and the necessity of conservative treatment or urgent abdominal laparotomy for HPVG depends on the specific patient conditions. Frequent monitoring (abdominal signs, CT and laboratory examination) are good for discovering disease changes and predicting patient prognosis. At the same time, abdominal sign and laboratory inspection are also important means to evaluate whether the patients have intestinal ischemic necrosis.

## Abbreviations

CT: computed tomography
HPVG: Hepatic portal vein gas
N%: neutrophilic granulocyte percentage
WBC: white blood cell
